# Evaluation of PD-L1 and other immune markers in bladder urothelial carcinoma stratified by histologic variants and molecular subtypes

**DOI:** 10.1038/s41598-020-58351-6

**Published:** 2020-01-29

**Authors:** Huili Li, Qingzhao Zhang, Lauren Shuman, Matthew Kaag, Jay D. Raman, Suzanne Merrill, David J. DeGraff, Joshua I. Warrick, Guoli Chen

**Affiliations:** 1 Department of Pathology, Penn State College of Medicine, Hershey, PA USA; 2 Department of Biochemistry and Molecular Biology, Penn State College of Medicine, Hershey, PA USA; 3 Department of Surgery, Penn State College of Medicine, Hershey, PA USA; 4 Department of Laboratory Medicine, Geisinger Medical Center, Danville, PA USA

**Keywords:** Biomarkers, Bladder

## Abstract

Although advanced bladder cancer overall has a poor prognosis, a subset of patients demonstrate durable response to immune checkpoint inhibitors. Evidence shows that the response to checkpoint inhibitors may be associated with type and degree of immune infiltration in the tumor microenvironment. Here, we evaluated immune markers stratified by molecular subtypes and histologic variants. The study utilized a series of urothelial carcinomas (UCs) by tissue microarray, on which histologic variants and molecular subtypes had previously been established. PD1, CD3, CD8 and CD68 expression was evaluated by immunohistochemistry in tumor infiltrating immune cells, while PD-L1 expression in the tumor microenvironment was assessed. Each marker was scored semi-quantitatively (score 0–3). Tumors were clustered by marker scores using agglomerative methods, and associations among markers, histologies, and molecular subtypes were analyzed. PD-L1 expression in the tumor microenvironment significantly correlated with presence of CD3, CD8 and chronic inflammation. Urothelial carcinoma may be classified as either immune high or low based on marker expression. The immune high group is enriched in higher CD3, PD-L1, and genomically-unstable molecular subtype, suggesting it may respond to checkpoint inhibitors. We also identified a degree of intratumoral heterogeneity in immune markers in bladder cancer.

## Introduction

The overall survival rate in bladder cancer patients with metastatic disease is suboptimal and has not significantly changed in the past several decades^1,2^. Immune checkpoint inhibitor (ICI) therapy has recently demonstrated promising anti-tumor effects. At present, there are five Food and Drug Administration (FDA) approved PD1/PD-L1 inhibitors for advanced urothelial carcinoma (atezolizumab, pembrolizumab, nivolumab, durvalumab, and avelumab)^3^. Because the response rate of urothelial carcinoma (UC) to ICIs is 15–21% in unselected metastatic patients, identification of the potentially responsive subpopulation is critical. Currently, predictive biomarkers that have been extensively studied and used in clinical and/or scientific settings predominantly include expression of PD-1 and PD-L1^4–7^, tumor inflammatory microenviroment^8^ and tumor mutational burden (TMB)^9^. The FDA has approved companion PD-L1 diagnostic tests in several tumor types. However, each companion test has been developed with its own reagents, working conditions and interpretation criteria^3^. The subjective scoring system, as well as non-comparable and inconsistent PD-L1 staining companion tests limit their predictive capability. Meanwhile, given the relatively complicated and extremely dynamic nature of tumor immune responses, the concept of immunoscore has been proposed. Simply, it is defined by evaluating the tumor immune microenvironment through various immune markers such as CD3, CD8, CD68, PD1, and PD-L1 in tissue sections in lung and colorectal cancer^10–12^, and potentially offers a relatively uniform parameter of prognostic and predictive values.

UC is among the most biologically and histologically diverse cancers. In addition to conventional morphology, UC can include pure or mixed elements of squamous differentiation, glandular differentiation, nested, plasmacytoid, sarcomatoid and/or rarer variants^13,14^. More recent studies have also subtyped UC based on molecular features, such as basal and luminal types by transcriptional profiling^15–18^. The consensus molecular classification of muscle-invasive bladder cancer, was published by Kamoun *et al*., 2018, and identified a consensus set of six molecular classes: Luminal Papillary (24%), Luminal NonSpecified (8%), Luminal Unstable (15%), Stroma-rich (15%), Basal/Squamous (35%), and Neuroendocrine-like (3%)^19^. Additional global mRNA profiling and molecular pathology studies from the Lund University group demonstrated five tumor-cell phenotypes: Urothelial-Like, Genomically-Unstable, SCC-like, Mesenchymal-like and Small-Cell/Neuroendocrine-like^20^. Specific subtypes are enriched in specific histologic variants. Certain histologic variants tend to be aggressive.

Interestingly, a higher response rate to atezolizumab was observed in luminal cluster II subtype compared to luminal cluster I, basal cluster I, and basal cluster II groups according to The Cancer Genome Atlas (TCGA) molecular subtyping system^21^, although another study showed basal cluster I subtype with the highest response rate to nivolumab^22,23^. Defined immune cell gene expression profiling showed correlation with response to subgroups of nivolumab and pembrolizumab trials^22^. Therefore, understanding relationships among the tumor immune microenvironment, molecular subtypes and histologic variants may help to develop a better method predicting responses to ICIs.

In this study, we evaluated the expression of PD1, PD-L1, CD3, CD8 and CD68, and chronic inflammation in a series of UCs by tissue microarray including 77 cases of carcinoma *in situ* (CIS), 40 non-invasive papillary urothelial carcinoma (NIPUC), and 143 invasive UCs, including conventional UC and six histologic variants^24–26^. Half of the cases had been assigned a molecular subtype in a prior study, using the Lund University approach^24–26^. The aims of our study were to explore the possibility of using multi-parameter biomarkers for immunotherapy response prediction, and facilitate understanding the immune characteristics based on histologic variants and molecular subtypes in UC.

## Results

### Immune high and immune low clusters of UC

In this study, the overall lymphocytic infiltration is interpreted as chronic inflammation. The level of chronic inflammation, expression of PD1 and PD-L1, and biomarkers of total T lymphocytes (CD3), cytotoxic “effector” T cells (CD8) and tumor-associated macrophages (CD68) were evaluated using a scoring system as described in *Material and methods*. Representative results following IHC for CD3, CD8, CD68, PD1 and PD-L1, as well as chronic inflammation, are shown in Supplementary Fig. S1.

**Figure 1:**
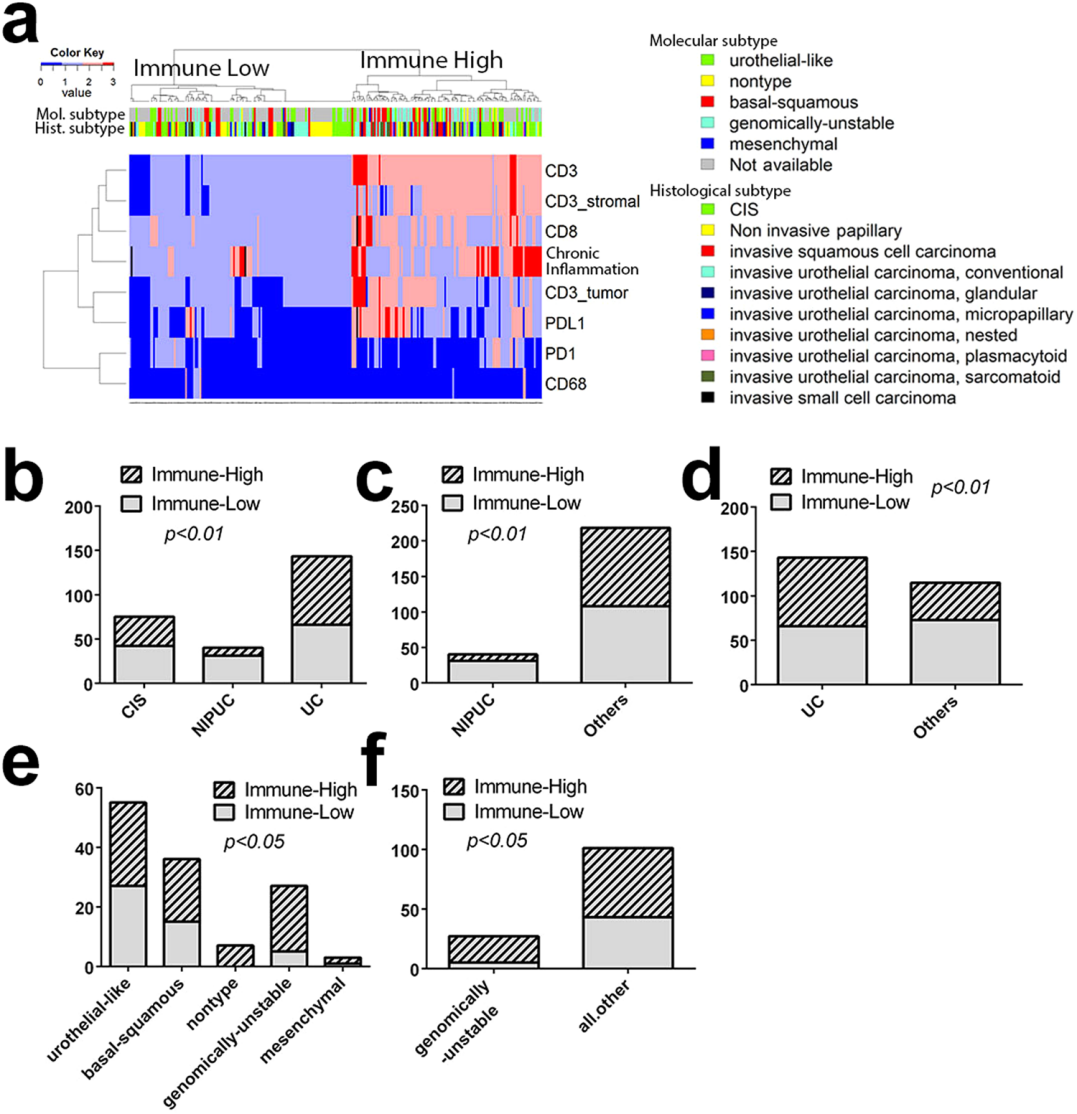
Immune marker score analysis in association with histological variants and molecular subtypes. (**a**) Unsupervised hierarchical clustering of all UC cases. Each row is a marker and each column is a patient. Top bar indicates histological variants and molecular subtypes. (**b**) Distribution of histological variants (CIS, NIPUC, and invasive UC) and immune high and immune low clusters (*Chi*-square test, *p* < 0.01); (**c**) NIPUC is significantly associated with immune low cluster (Fisher’s exact test, *p* < 0.01); (**d**) Invasive UC is significantly associated with immune high cluster (Fisher’s exact test, *p* < 0.01); (**e**) Distribution of molecular subtypes (urothelial-like, basal-squamous, nontype, genomically-unstable, mesenchymal) in immune high and immune low clusters (*Chi*-square test, *p* < 0.05); (**f**) Genomically-unstable subtype is significantly associated with immune high cluster (Fisher’s exact test, *p* < 0.01).

In an effort to identify biomarkers closely related to PD-L1 expression and thus potentially used as a supplement to predicting responses to ICIs, we performed unsupervised hierarchical clustering based on assigned scores following IHC using our panel of tested markers (CD3, CD8, CD68, PD1, PD-L1 and chronic inflammation). We found that CD3, and CD8 scores were individually moderately correlated with PD-L1 scores (Spearman’s rank-order correlation; r = 0.58 for CD3; 0.46 for CD8; p < 0.0001) analysis, but only weakly correlated with CD68 and PD1 (Spearman’s rank-order correlation; r = 0.16 for CD3; 0.23 for CD8; p < 0.01). PD-L1 scores also seem weakly associated with intra-tumoral CD3 in the dendrogram (Fig. 1a).

There appeared to be two clusters with distinct immune marker score patterns in similar sizes: immune high cluster and immune low cluster (Fig. 1a). The immune high cluster (n = 119) was enriched in specimens exhibiting higher in CD3, CD8, PD-L1 expression, and greater chronic inflammation. To elucidate the immune properties of invasive UC variants, unsupervised hierarchical clustering was also performed with invasive UC only. Similar immune high and low clusters were identified (Supplementary Fig. S2).

### Immune clusters are associated with specific histological variants

The distributions of immune high and immune low clusters in CIS, NIPUC and invasive UC groups were compared by Chi-square test (Fig. 1b) and Fisher’s exact test. Our results demonstrated that NIPUC was significantly enriched in the immune low cluster (Fig. 1c; *p* = 0.001, Fisher’s exact test). Invasive UC is significantly associated with the immune high cluster (Fig. 1d; *p* = 0.0059, Fisher’s exact test) compared to non-invasive tumors. Within the invasive UC variants, squamous and sarcomatoid histologies tended to be immune high compared to the other variants, but this was not statistically significant by *Chi-*square test (Supplementary Fig. S3).

### Immune high cluster is associated with genomically-unstable molecular subtype

**Figure 2:**
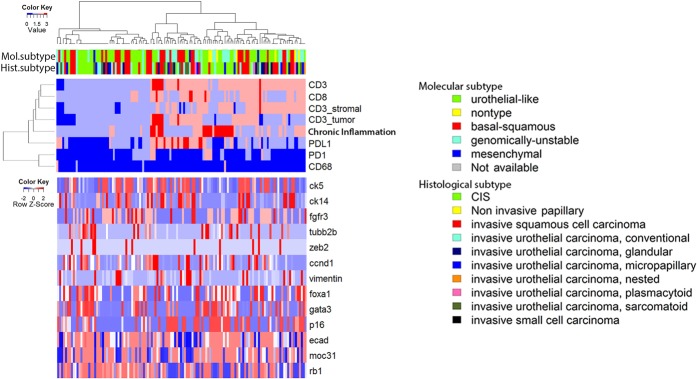
Supervised hierarchical clustering with immunohistochemistry staining of 13 proteins representing molecular subtyping genes demonstrates that the immune high and immune low clusters are not associated with the individual gene signatures. Only specimens with available data of the immunohistochemical stains are used for the hierarchical clustering. The dendrogram is determined by unsupervised hierarchical clustering of the immune markers (top panel). Expression levels of the 13 genes are plotted in the bottom panel following the same dendrogram (supervised).

Molecular subtyping schema from Lund University^27^ by immunohistochemical staining of 13 proteins as gene signatures (FOXA1, GATA3, CDH1, CCND1, P16, RB1, KRT14, KRT5, EPCAM, TUBB2B, Vimentin, ZEB2, FGFR3) were previously used to subtype our cohort^26^. To explore a potential association between molecular subtype defined by the Lund taxonomy and the immune high/low clusters identified by us, we performed supervised hierarchical clustering. As shown in Fig. 2, immune clusters did not clearly follow any individual marker of molecular subtype. The distribution of molecular subtypes in immune high and low clusters are also investigated. Tumors of genomically-unstable molecular subtype were significantly enriched in the immune high cluster (*p* = 0.0255, Fisher’s exact test) (Fig. 1e,f).

### Individual immune score distribution with histological variants

**Figure 3:**
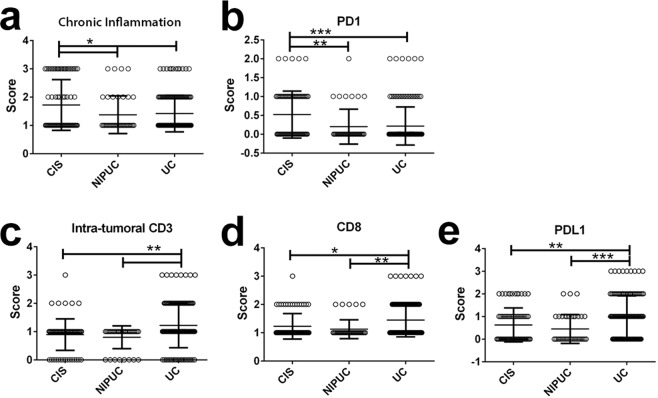
Comparisons of immune marker scores in CIS, NIPUC and invasive UC. (**a**,**b**) CIS has significantly higher chronic inflammation and PD1 scores as compared to NIPUC and invasive UC; (**c**–**e**) Invasive UC has significantly higher CD3, CD8 and PD-L1 scores as compared to NIPUC and invasive UC. All statistical significance was calculated by one with ANOVA with posttest as described in methods and materials and plotted as Mean ± SD. *p* < 0.05 is considered significant as *.

**Figure 4:**
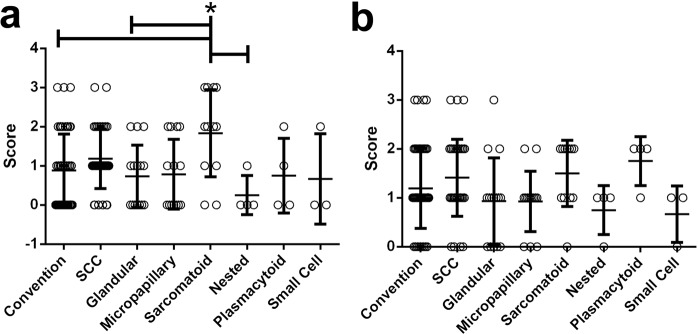
Immune scores of PD-L1 and CD3 among invasive UC variants. (**a**) Sarcomatoid variant shows significantly higher PD-L1 score as compared to the nested, glandular, and conventional UC. (**b**) Squamous, sarcomatoid, and plasmacytoid variants tend to have higher intra-tumoral CD3 scores. All statistical significance was calculated by one with ANOVA with posttest as described in methods and materials, and plotted as Mean ± SD. *p* < 0.05 is considered significant as *.

Analyzing scores of individual immune markers from histological variants demonstrated that CIS had significantly greater chronic inflammation (Fig. 3a) and PD1 expression (Fig. 3b) than NIPUC and invasive UC (*p* < 0.05). Invasive UC showed higher lymphocytic CD3 (Fig. 3c), CD8 (Fig. 3d), and PD-L1 (Fig. 3e) expression than CIS and NIPUC (*p* < 0.05). As the invasive UC was further stratified by histologic variants, sarcomatoid histology demonstrated a significantly higher PD-L1 score as compared to nested, glandular, and conventional histologies (Fig. 4a). Although being not statistically significant, invasive squamous, sarcomatoid and plasmacytoid histologies tended to have more intra-tumoral lymphocytes (Fig. 4b).

### Invasive UC demonstrated moderate intra-tumoral immune heterogeneity

**Figure 5:**
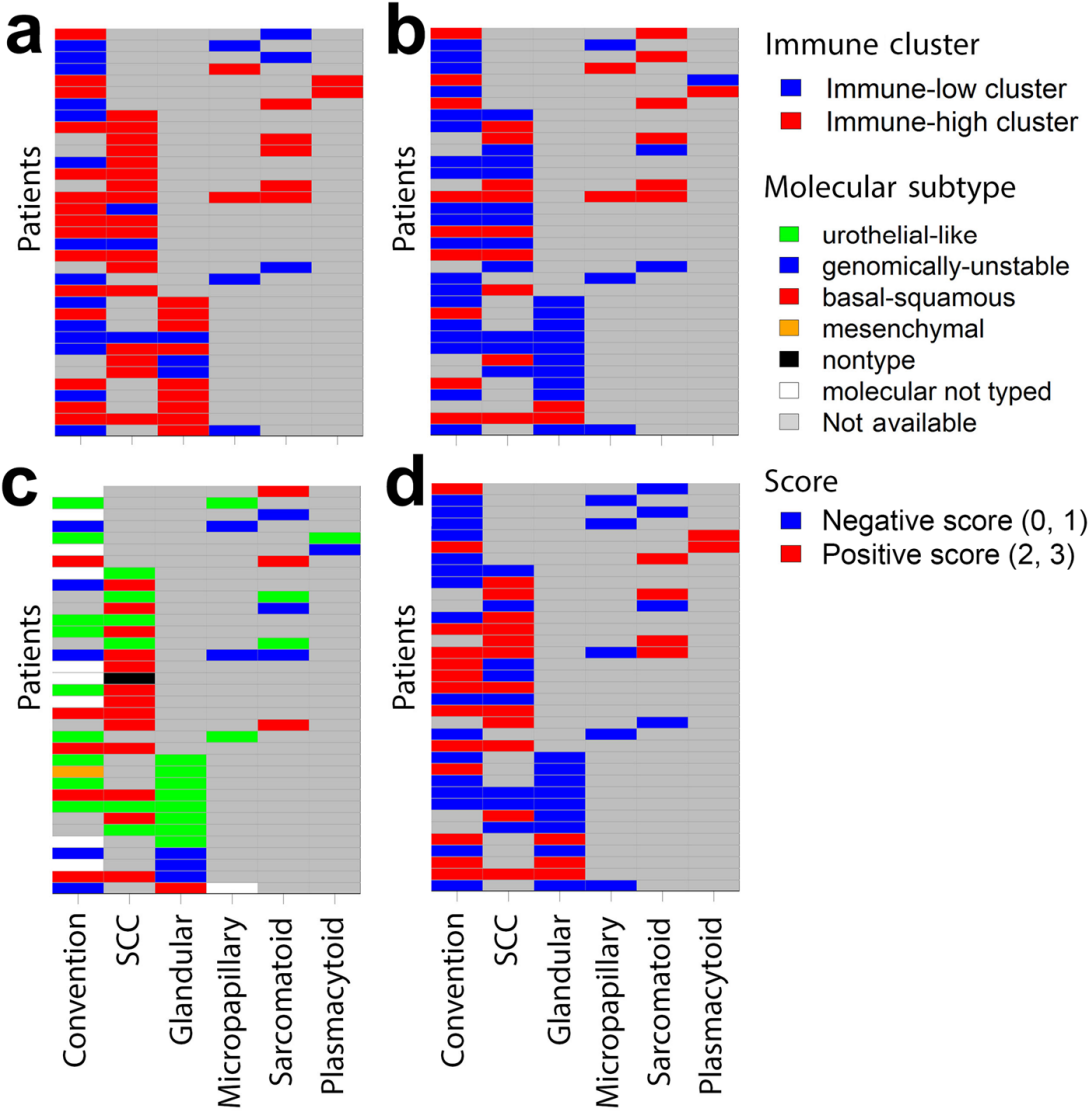
Intra-tumoral immune heterogeneity in invasive urothalial carcinoma. Each row represents a patient and each column is a histologic variant. (**a**) Distribution of immune low and immune high cluster profile in different histologic variants from the same patient. (**b**) Distribution of PD-L1 scores in different histologic variants from the same patient. (**c**) Distribution of molecular subtypes in different histologic variants from the same patient. (**d**) Distribution of intra-tumoral CD3 scores in different histologic variants from the same patient.

Within our TMA specimens, thirty-five patients with two or more distinctive histologies/variants were identified. To investigate intra-tumoral heterogeneity, immune clusters (low and high), molecular subtypes, and PD-L1 score, and intra-tumoral CD3 score were plotted in relationship to the histologic variants (Fig. 5). To further simplify the scoring system, score 0 & 1 are defined as negative score, while score 2 & 3 are defined as positive score. We found that 16 out of the 35 patients demonstrated consistent immune high features among the intra-tumoral histologic types (Fig 5a), 5 were consistently in immune low cluster, while the rest 14 demonstrated inconsistent immune clustering (Fig. 5a). Similar patterns of intra-tumoral heterogeneity in PD-L1 score (Fig. 5b), molecular subtype (Fig. 5c), and intra-tumoral CD3 score (Fig. 5d) were also observed.

## Discussion

UC is a group of morphologically and genomically diverse tumors, and these attributes have prognostic and therapeutic significance. With the recent development of check point immunotherapy, immunologic features of UC have caught intensive attention. In this study, we evaluated biological characteristics and expression patterns of various immune markers, stratified by histological and molecular features. Our findings further support the observation that certain histological variants and molecular subtypes tend to be more immunogenic, and therefore may be more likely to respond to immunotherapy. Also, intra-tumoral immune heterogeneity does exist and may contribute to treatment response.

UC is well recognized as an immunogenic and immunoresponsive tumor. Intravesicle bacillus calmette-Guerin (BCG) has been widely used in non-muscle-invasive bladder cancer for more than 40 years with superior efficacy^28,29^. Genome wide studies have also demonstrated that UC has the fourth highest overall mutational burden, which can consequently triggers immune responses^28^. Also, the tumor-associated lymphocytic infiltration may be related to clinical outcomes in UC^30^. As one of the tumor mechanisms to evade immune surveillance and promote immune tolerance, expression of immune checkpoint proteins such as PD1/PD-L1 are upregulated in tumor microenvironment following chronic antigen exposure^31^. In relation to this, targeting therapies with ICIs have demonstrated promising efficacy in multiple advanced cancers including UC. The detection of PD1/PD-L1 expression has been adapted in the companion tests to predict response to ICIs. As expected, PD-L1 score in tumor microenvironment (both tumor cells and tumor infiltrating immune cells) was significantly correlated with active immune markers (CD3, CD8,and chronic inflammation) in this study, which is consistent with the idea that immune tolerance is associated with immunogenic microenvironment^32^. Unexpectedly, the PD1 positivity was minimum through all the specimens even with appropriate positive and negative controls.

Tumor cells closely interact with its microenvironment and elicit immunoediting to escape immune attacks^32,33^. Based on the chronic inflammation and PD-L1 expression, the microenvironment can be divided into immunogenic (enriched in immune cells, “hot”) and non-immunogenic (lack of immune markers, “cold”) categories, which have been suggested in multiple studies as an indicator for immunotherapy response^32,34^. Immunogenic (“hot”) microenvironment is believed exhibit a better response to immunotherapy. Clinical trials and research studies have also been designed to augment the immunogenicity by combining agents that can enhance immune responses, such as histone deacetylase inhibitors and DNA demethylation agents, etc^32,35–39^. Our study provides more evidence that different pathologic features, histological variants and molecular subtypes of UC possess different immunogenicity. In particular, we found that NIPUC are significantly enriched in immune low cluster, while high grade invasive UC cases are significantly enriched in immune high cluster with higher CD3, CD8 and PD-L1 scores implying higher immunogenicity. Studies on tumor infiltrating lymphocytes (TIL) have shown that TIL density positively correlates with grade, and the prognostic effect of TILs depends on stage^40–42^. Although CIS showed significant higher chronic inflammation, a significantly higher PD1 score was also observed, which might counter balance the immune activation and contribute to an immune ‘neutral’ status. So compared to those with innate “hot” tumor microenvironment characteristics such as invasive UC variants, CIS and NIPUC may require additional combination therapy to boost responses to ICIs if the treatment is considered.

In addition, we studied the relation of molecular subtypes with immune clusters and showed that genomically-unstable subtype is significantly enriched in immune high cluster. Genomically-unstable subtype, comprising about 24–38% of invasive UC, was identified via gene expression profile by Lund University group^20,27^, and is characterized by loss of RB1 and high genomic instability^20,27^. According to Sjodahl *et al*., 2012, this subtype coincides with the previous published high risk UC variant^43^. Furhter, the genomically-unstable subtype may have the highest response rate to immune checkpoint blockage^15,23^, which is in keeping with the hypothesis that immune high cluster might have better response to the immunotherapy. The behind mechanisms, for example, whether this molecular subtype is closely related the group of tumors with high tumor mutational burden or its decreased cell adhesion and lack of tissue organization along the tumor-stroma interface, etc, remain unclear and need more studies. Nevertheless, the molecular subtyping may be used as a part of predictive mode to the immunotherapy. Additionally, the agreement of our results and other publications also provide certain assurance for the major concern on TMAs’ sampling size. Though validation in larger cohorts with surgical specimen will be needed in the future studies.

We have previously explored the potential relationship between histological variants and molecular subtypes^26^ and reported that invasive UC often has intra-tumoral molecular heterogeneity. However, low frequency of intra-tumor molecular heterogeneity in UC has also been reported^44^. In this study, we compared the immune profiles of different histologic variants in each of the patients with two or more distinctive histologies. Interestingly, we found that about 60% of patients showed consistent immune profile with multiple TMAs, suggesting the idea that UC has moderate immune heterogeneity. However, about 40% of the patients demonstrated intra-tumoral heterogeneity with inconsistent immune features given of histologic features. Due to the size limitation of TMAs, full surgical specimen might be able to provide more consistent and accurate information. So extensive and adequate sampling should be necessary for an adequate evaluation of immune features in UC.

In summary, there are two main findings in our study. First, UC has moderate intra-tumoral immune heterogeneity, perhaps associated with its histologic and molecular heterogeneity, and therefore sampling adequacy will be critical if the immune features are evaluated for the prognostic and predictive purposes. Second, UC can be classified as immune high and immune low clusters, which may be associated with the tumor invasive status, molecular subtypes and histologic phenotypes. This finding will provide more information for guiding immunotherapy strategies in UC patients. In conclusion, our study adds more information to understand the relationship of immunomarkers, histologic variants and molecular subtypes in UC, which may lead to better strategies in management of this group of patients.

## Material and Methods

### Tissue microarray (TMA)

Tissue microarrays (TMAs) had previously been constructed from cystectomy specimens, as reported^24,25^. Briefly, histologically and spatially (if within the same patient) distinct specimens were selected by the GU pathologist for inter- and intra-tumor comparisons with most specimens being of tumor stage pT2 or above. TMAs were constructed in the pathology research core at Penn State Health Milton S. Hershey Medical Center via standard procedures^24,25^. Histology and Molecular subtype case numbers are summarized in Supplementary Table S1.

### Immunohistochemistry (IHC) assays

TMA sections (5 μm in thickness) were used for IHC. PD1 and PD-L1 IHC were performed at the Pathology research core of Hershey Medical Center using Discovery XT Autostainer from Ventana. CC1 (Ventana #950–500) was used for antigen retrieval. Primary antibodies for PD1 (Ventana #760–4895) and PD-L1 (Ventana #740–4859) were used after optimization with serial titrations. Briefly, antigen retrieval was performed with CC1 for 48 minutes, then primary antibodies were incubated for 16 minutes. Amplification kit (anti-rabbit HQ (Ventana #760–4815, 8 minutes), anti HQ HRP (Ventana #760–4820, 8 minutes), Disc Amp kit (Ventana, #760–052) and Disc anti-HQ HRP (Ventana, #760–4602)) was used for PD-L1 detection. Secondary anti-mouse (Ventana, #760–4310) was used for PD1 detection. All slides were treated by machine with Chromomap DAB kit (Ventana #760–159), then counterstained with Hematoxilyn II (Ventana #790–2208), and followed by bluing reagent (Ventana #760–2037). Positive and negative controls are included in all IHC stains. All other IHC was performed by manual methods as previously described^45^. Briefly, slides were deparaffinized and rehydrated through a series of graded alcohols and washed in deionized water for 3 minutes. Antigen retrieval was performed by placing slides in 1% antigen unmasking solution (Vector Labs, Burlingame, CA) and heating slides for 20 minutes on high power in a pressure cooker (Cuisinart CPC-600, Conair Corporation, Stamford, CT). Steam was released in short bursts to prevent boiling and to preserve tissue integrity. Slides were cooled to room temperature and washed for three 10-minute washes in phosphate-buffered saline (PBS; pH 7.4). All incubations were performed at room temperature unless otherwise noted. Endogenous peroxidases were blocked by incubation in 1% hydrogen peroxide in methanol for 20 minutes, and slides were again washed for three 10-minute washes in PBS. Sections were incubated in PBS containing horse serum (Vector Labs) for 1 hour to reduce nonspecific antibody binding and then incubated overnight with primary antibody at 4 °C in a humidified chamber. Primary antibodies for CD3 (mouse 1:200; M7254, Dako), CD8 (mouse 1:50; M7103, Dako), and CD68 (mouse 1:1,500; M0718, Dako) were used as described by Sjodahl, *et al*.^42^. Following overnight incubation, slides were washed in three 10-minute PBS washes, and sections were incubated in biotinylated secondary antibody diluted in PBS containing horse serum (1:200; Vector Labs) for 1 hour. Specific antibody binding was visualized using Vectastain Elite ABC Peroxidase kit (Vector Labs) according to the manufacturer protocol with diaminobenzidine (DAB) substrate buffer as the chromogen (Thermo Fisher Scientific, Waltham, MA).

### Immunohistochemistry evaluation

Each marker was scored based on published scoring system^3,10,12^ and information of histologic subtypes was blinded to pathologists during evaluation. In short, IHC stains were graded 0 to 3 (No stain to diffuse stain) initially by Dr. Li and then confirmed by Dr. Chen. Both pathologists are blinded to the histologic variants and molecular subtypes during evaluation. Scores for H&E, CD8, CD68, PD1, are based on overall geographyically immunostaining distribution in lymphocytes, without distinguishing tumor versus stroma. CD3 staining was evaluated as overall CD3, intra-tumoral CD3 (tumor area with/without thin desmoplastic stromal) and stromal CD3 (well-demarcated stromal area). According to the published Ventana IHC assay method with the SP142 antibody (Ventana, AZ, USA), PD-L1 positivity was defined by both PD-L1 expression on tumor cells and tumor infiltrating immune cells (including macrophages, dendritic cells, and lymphocytes)^3^. Negative PD-L1 was defined as scores 0 and 1, similar to prior study^3^, while scores 2 and 3 (≥5% of positivity) are defined as positive (see Table S2). Chronic inflammation is scored by evaluating the overall levels of lymphocytic infiltration with routine H&E staining to provide additional information of tumor-immune response besides specific IHC markers. The detailed scoring system for each antibody is listed in Supplementary Table S2.

### Statistical analysis

The expression patterns of the immune marker were analyzed with associated histologic variants and molecular subtypes by R statistical software (http://www.r-project.org) with unsupervised hierarchical clustering packages and customized routines^35,36^. Contingency test, one-way ANNOVA with Turkey post-test and Spearman correlation are used for *p*-value calculation. *P* < 0.05 is considered significant.

This research including all the experimental protocols and use of patient specimens was approved by The Human Subjects Protection Office at Penn State College of Medicine and all methods were carried out in accordance with the relevant guidelines and regulations. All subjects were 18 years old or greater, and the Institutional Review Board (IRB) issued a waiver of consent.

## Supplementary information

supplemental tables and figures.
