# Supplementary material for: Evaluation of PD-L1 and other immune markers in bladder urothelial carcinoma stratified by histologic variants and molecular subtypes
Source: Sci Rep. 2020 Jan 29;10:1439. doi: 10.1038/s41598-020-58351-6 (PMC6989654; doi:10.1038/s41598-020-58351-6)
Supplement: Supplementary file 1 — supplemental tables and figures. [file 41598_2020_58351_MOESM1_ESM.pdf]

Evaluation of PD-L1 and other immune markers in bladder urothelial carcinoma stratified by histologic variants and molecular subtypes

Huili Li[1], Qingzhao Zhang[1], Lauren Shuman[2], Matthew Kaag[3], Jay D. Raman[3], Suzanne Merrill[3] David J. DeGraff[1,2,3], Joshua I. Warrick[1,3#], Guoli Chen[1, 4#].

[1]Department of Pathology, Penn State College of Medicine, Hershey, PA; [2]Department of Biochemistry and Molecular Biology, Penn State College of Medicine, Hershey, PA; [3]Department of Surgery, Penn State College of Medicine, Hershey, PA. [4]Department of Laboratory Medicine, Geisinger Medical Center, Danville, PA. #co-corresponding authors

[#]Co-corresponding Authors

Joshua I. Warrick, MD
Department of Pathology
Milton S. Hershey Medical Center
Penn State College of Medicine
500 University Drive
Hershey, PA 17033
email: jwarrick@pennstatehealth.psu.edu

Guoli Chen, MD, PHD
Department of Laboratory Medicine
Geisinger Medical Center
100 N Acedemy Aveune
Danville, PA 17822
email: gchen1@geisinger.edu

Supplementary Table. S1. Summary of histologic variants and molecular subtypes in this study.

CIS	77
NIPUC	40
UC, convention	52
UC, glandular	15
UC, micropapillary	14
UC, scc	39
UC, sarcomatoid	12
UC, nested	4
UC, plasmacytoid	4
UC, small cell	3
Basal-squamous	36
Urothelial-like	55
Genomically-unstable	27
mesenchymal	3
nontype	7

Supplementary Table. S2. Summary of scoring system for each marker by H&E and IHC stains.

Immune Markers	***IHC target***	***Function***	***Scoring System***			
			0	***1***	***2***	***3***
**HE**	lymphocytes	Chronic inflammation		<10% area	10-49% area	≥50% area
**CD3**	T cell	Immune response	no stain	<50% area, weak	<50% area, strong or ≥50% area, moderate	≥50% area, strong
**CD8**	Cytotoxic T cell	Immune response	no stain	<50% area, weak	<50% area, strong or ≥50% area, moderate	≥50% area, strong
**CD68**	Macrophage/monocytes	Immune suppression	no stain	<50% area, weak	<50% area, strong or ≥50% area, moderate	≥50% area, strong
**PD1**	Predominantly Lymphocytes	Immune suppression	no stain	<5% area	5-49% area	≥50% area
**PDL1**	Predominantly Tumor cells	Immune suppression	no stain	<5% area	5-49% area	≥50% area

Supplementary Figure. S1. Representative H&E and immunohistochemically (IHC) stained sections for various markers. (A-F: 100x). The sections were evaluated by criteria descripted in Supplementary Table S2. A. H&E stain, score=1 for chronic inflammation; B. IHC stain, score=2 for CD3; C. IHC stain, score=2 for CD8; D. IHC stain, score=0 for CD68; E. IHC stain, score=1 for PD1; F. IHC stain, score=3 for PD-L1.

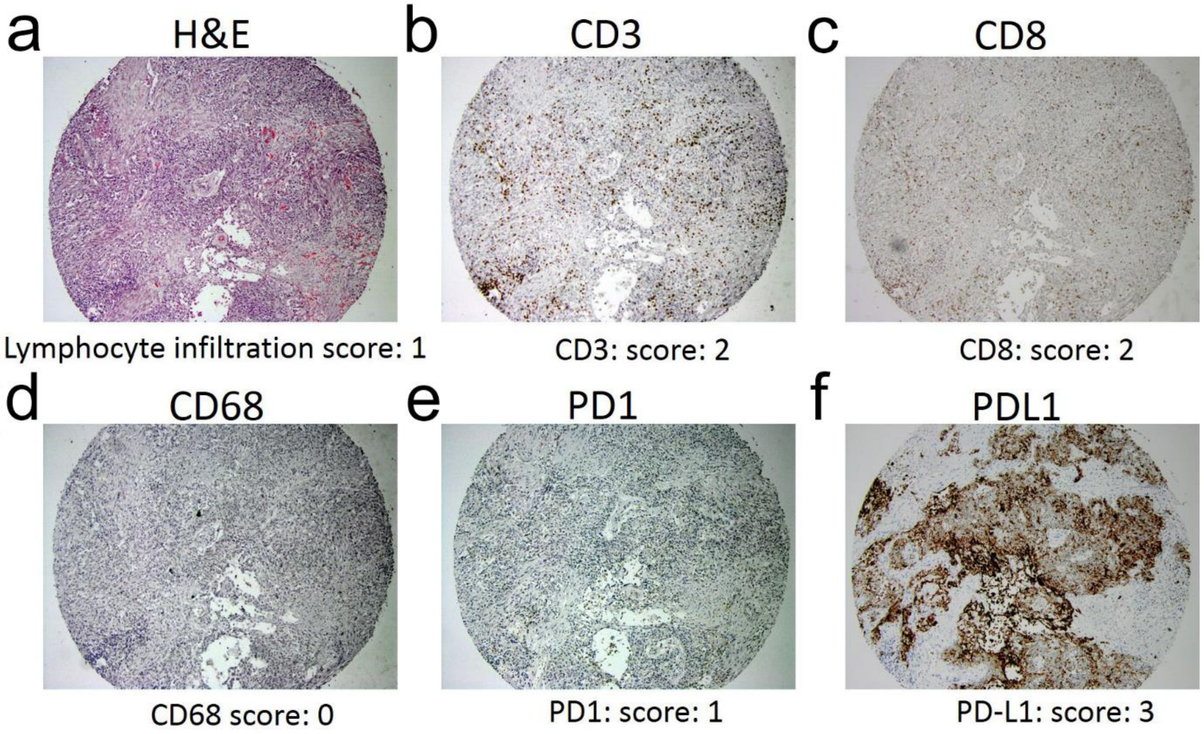

Supplementary Figure. S2. Immune high and immune low clusters in invasive UC by unsupervised hierarchical cluster. The same method from Figure 2 is applied to only invasive UC specimens. A similar immune high and immune low clustering pattern is identified.

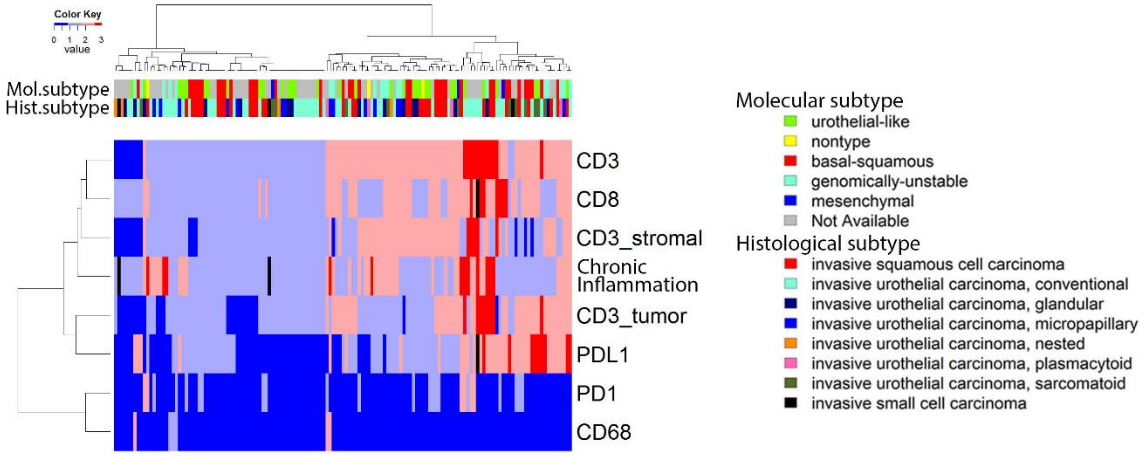

Supplementary Figure. S3. Distribution of histologic variants of invasive UC in immune high and immune low clusters. The molecular subtypes and histology subtypes in the cluster dendrogram from spl. Fig. 1 are analyzed and compared. Within the invasive UC variants, sarcomatoid and squamous histologies show more case numbers in the immune high cluster, as compared to the other variants, but without statistical significance.

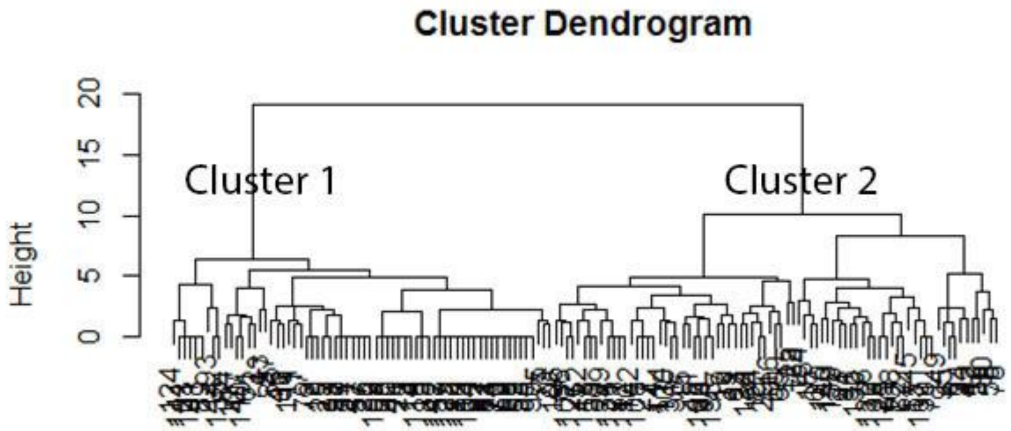

	cluster1-66 specimen		cluster2-77-specimen	
Molecular subtypes	urothelial-like	15	urothelial-like	17
	basal-squamous	15	basal-squamous	21
	nontype	1	nontype	2
	genomically-unstable	5	genomically-unstable	14
	mesenchymal	1	mesenchymal	2
Histology subtypes	convention	28	convention	24
	SCC	16	SCC	23
	glandular	7	glandular	8
	micropapillary	7	micropapillary	7
	sarcomatoid	3	sarcomatoid	9
	nested	3	nested	1
			plasmacytoid	4
	smallcell	2	smallcell	1
